# Changes in Acupuncture-Induced Specific Acupoint Neurotransmitters are Possibly Related to Their Physiological Functions in Rats

**DOI:** 10.1155/2023/4849528

**Published:** 2023-02-21

**Authors:** Huong Thi Mai Nguyen, Der-Yen Lee, Chung-Hsiang Liu, Ching-Liang Hsieh

**Affiliations:** ^1^Graduate Institute of Acupuncture Science, China Medical University, Taichung, Taiwan; ^2^Graduate Institute of Integrated Medicine, China Medical University, Taichung, Taiwan; ^3^Department of Neurology, China Medical University Hospital, Taichung, Taiwan; ^4^Department of Chinese Medicine, China Medical University Hospital, Taichung, Taiwan; ^5^Chinese Medicine Research Center, China Medical University, Taichung, Taiwan

## Abstract

This study investigated changes in neurotransmitters induced by the application of electroacupuncture (EA) at Zusanli (ST36) and Neiguan (PC6). A total of 30 rats were divided into five groups: sham, ST (EA at bilateral ST36 and ST37), ScT (ST plus previous neurectomy of the bilateral sciatic nerves), ScS (sham plus previous neurectomy of the bilateral sciatic nerve), and PC (EA at bilateral PC6 and PC7). The P2X2 receptor expression was stronger in the sham group than in the ST and PC groups (both *p* < 0.05) but similar between the sham and ScT groups (*p* > 0.05). Dopamine levels in the extracellular fluid surrounding the acupoints were higher in the PC group than in the sham and ST groups during the postacupuncture period (both *p* < 0.05). Glutamate levels in the extracellular fluid surrounding the acupoints were higher in the ST group than in the sham group during the acupuncture period (*p* < 0.05) and higher in the ST group than in the sham and PC groups during the postacupuncture period (both *p* < 0.05). Serum adrenaline and noradrenaline levels were higher in the PC group than in the sham, ST, and ScT groups (all *p* < 0.05). Glutamate levels in the CSF were higher in the ST group than in the sham, ScS, and PC groups (all *p* < 0.05). GABA levels in the CSF were higher in the ST group than in the sham, ScT, and PC groups (all *p* < 0.05). EA at ST36 and ST37 and PC6 and PC7 exerted an analgesic effect, EA at PC6 and PC7 can enhance heart function, and EA at ST36 and ST37 modulates the cerebral cortex. However, the study needs an evaluation of direct pain behavior, heart function, and brain function in the future.

## 1. Introduction

Acupoints and meridians are the main components of acupuncture. Our previous study reported the specificity of known acupoints; for example, electroacupuncture (EA) at PC6 can downregulate the production of the adrenaline hormone in the hippocampus and cerebral cortex, whereas EA at ST6 can upregulate its production [1]. Studies have performed the partial least square discriminant analysis to determine the distribution of metabolite profiles in the cerebral cortex, hippocampus, hypothalamus, heart, and stomach tissues of rats in sham, ST36, and PC6 groups [1, 2]. The findings of these studies indicated that acupuncture or EA at acupoints can exhibit the specificity of those acupoints; however, whether acupoint specificity can be observed in the environment surrounding those acupoints and drive different acupuncture signal transmission remains unclear.

Application of manual acupuncture to the tibialis anterior muscle increased the local extracellular concentrations of adenosine triphosphate, adenosine diphosphate, and adenosine, as determined through high performance liquid chromatography with the microdialysis technique [3]. A study examining dermal microdialysis data reported that the application of EA to Quze (PC3) at 10 Hz for 15 min increased nitric oxide and 3′, 5′ cyclic guanosine monophosphate levels in the Ximen (PC4), thus improving local microcirculation [4]. The findings indicated that the application of acupuncture at different acupoints can lead to the production of different substances or neurotransmitters in tissues surrounding the acupoints. These substances or neurotransmitters may be responsible for the effect of acupuncture. The application of manual acupuncture or EA at acupoints can activate sensory mechanoreceptors and nociceptive receptors in tissues surrounding the acupoints [5]. Most of the acupoints contain many nerve endings and Meissner's corpuscles, Ruffini endings, Pacinian corpuscles, and Krause end bulbs that are all encapsulated. The insertion of an acupuncture needle into an acupoint with Der qi can activate neural and neuroactive components; the collection of activated neural and neuroactive components in skin, muscle, and connective tissues surrounding the inserted needle is referred to as a neural acupuncture unit [6]. Acupuncture induces the release of ATP from the skin, and the released ATP binds to the purinergic receptor of sensory nerves and subsequently conveys the acupuncture signal from the spinal ganglia to higher centers including the spinal cord, brain stem, hypothalamus, and cerebral cortex [7, 8]. Taken together, purinergic receptors, sensory nerves, and mechanoreceptors play a crucial role in acupuncture signal transmission. In addition, acupuncture signal transmission includes circular and neural transmission [9]. To determine the specific physiological functions of acupoints for clinical application, the present study investigated changes in neurotransmitters released in muscle tissues surrounding the acupoints and in the extracellular fluid, peripheral blood, and cerebrospinal fluid (CSF) after the application of acupuncture at the Zusanli (ST36) and Neiguan (PC6).

## 2. Materials and Methods

The present study was to follow the ARRIVE reporting guidelines for experiment animals including species, sex, body weight, and group.

### 2.1. Animals

Male Sprague–Dawley (SD) rats, weighing 225–250 g, were purchased from BioLASCO Taiwan Co., Ltd. The rats were raised in a 12 h light/dark environment in the animal center of China Medical University (CMU). The room temperature and humidity were controlled using an air conditioner at 20°C–24°C and 50%–70%, respectively. The rats were provided with adequate drinking water and a fixed formulated diet. Animal use was approved by the Institutional Animal Care and Use Committee of CMU, and all animal experiments were performed in accordance with recommendations in the Guide for the Use of Laboratory Animals (National Academy Press). Protocol No. CMUIACUC 2020 279 1, CMUIACUC 2020 279 2, CMUIACUC 2020 279 3).

### 2.2. Groups

A total of 30 SD rats were randomly divided into five groups (*n* = 6 in each group), and that was according to the GraphPad random assignments. (1) In the sham group, acupuncture needles (No. 32 G, Quian Hui, New Taipei City, Taiwan; 0.27 mm in diameter and 13 mm in length) were applied to the subcutaneous bilateral ST36 and ST37 under 2% isoflurane anesthesia that were similar to “tong shen cun” in the human body without electrical stimulation for 30 min. (2) In the ST group, acupuncture needles were inserted into bilateral ST36 (cathode) and ST37 (anode) acupoints under 2% isoflurane anesthesia, and the needles were applied to an electrical stimulation machine (Trio 300, Japan); electrical stimulation was applied at 2 Hz until a slight muscle contraction was visible for 30 min. (3) In the ScT group, the same method was applied as that in the ST group; however, the bilateral sciatic nerves were cut proximal to the trifurcation 30 min prior to the insertion of acupuncture needles. (4) In the ScS group, the same method was applied as that in the sham group; however, bilateral sciatic nerves were cut proximal to the trifurcation 30 min prior to the insertion of acupuncture needles. (5) In the PC group, the same method was applied as that in the ST group; however, acupuncture needles were inserted into PC6 and PC7.

### 2.3. Sciatic Neurectomy

The rats were placed in the side-lying position under 2% isoflurane anesthesia. A longitudinal incision was made using a surgical scalpel from the pelvic cavity to the trifurcation of the tibial, common peroneal, and sural nerves to expose the sciatic nerve. The sciatic nerve was completely transected from proximal to the trifurcation. A sciatic neurectomy was performed 30 min prior to the insertion of acupuncture needles.

### 2.4. Acupuncture Acupoints

Zusanli (ST36) is located in the posterolateral aspect of the knee joint, approximately 5 mm below the capitulum fibulae. Shangjuxu (ST37) is located 5 mm down from the ST36 of the hind leg of the rats [10, 11]. Daling (PC7) is located in the middle of the wrist of the rat's paw. Neiguan (PC6) is located 2 mm above the transverse crease of PC7, between the tendons of the palmaris longus and flexor carpi radialis muscles, and penetrates the flexor digitorum superficialis [12, 13].

### 2.5. Collection of Extracellular Fluid, Blood, CSF, Muscle, Spinal Cord, and Brain Tissue Samples

**Figure 1:**
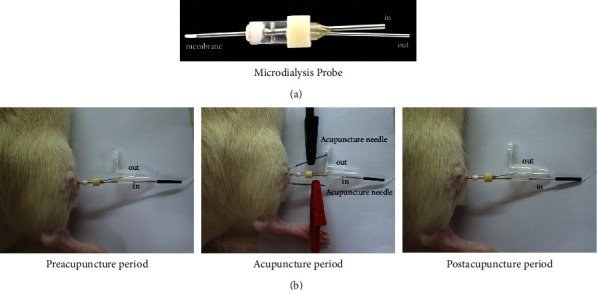
A photo case of rats during acupuncture operations. (a) Microdialysis probe is composed of an in tube, out tube, and membrane; (b) extracellular fluid microdialysis divided into preacupuncture period (before electroacupuncture treatment), acupuncture period (during electroacupuncture treatment), and postacupuncture period (after electroacupuncture treatment), each period was 30 min; black color: the acupuncture needle inserted into ST36 serves as a cathode; red color: the acupuncture needle inserted into ST37 serves as an anode.

First, the rats were anesthetized with 2% isoflurane. Subsequently, a needle was inserted into ST36 or PC6, and a microdialysis probe (Eicom, Japan) was implanted between ST36 and ST37 or between PC6 and PC7. The samples were collected 30 min after the implantation of the probe (baseline, preacupuncture period). Then, acupuncture needles were inserted into bilateral ST36 and ST37 or PC6 and PC7 (ipsilateral as a pair), and 2 Hz EA was applied for 30 min. The samples were collected simultaneously during the EA period (acupuncture period). EA stimulation was stopped, and the needles were removed. Subsequently, the samples were collected for 30 min (postacupuncture period). The perfusion rate was set at 1.5 *μ*L/min. The samples were placed in a box with ice cubes to prevent protease activation and then stored at −80°C until they were used for the metabolomic analysis of neurotransmitters (Figures 1(a) and 1(b)).

**Figure 2:**
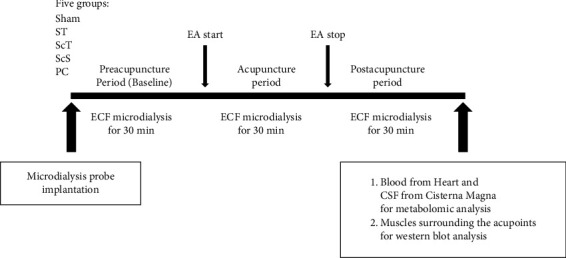
The flowchart of the experimental overview. First, a total 30 rats were divided randomly into five groups of sham, ST, ScT, ScS, and PC; second, the microdialysis probe was implanted and extracellular fluid (ECF) microdialysis for 30 min (preacupuncture period and baseline); third, inserting acupuncture needles with electroacupuncture treatment (EA start) and ECF microdialysis for 30 min (acupuncture period); and fourth, taking acupuncture needles out (EA stop) and ECF microdialysis for 30 min (postacupuncture period); finally, blood from heart and cerebrospinal fluid (CSF) from cisterna magna for metabolomic analysis and muscles surrounding the acupoints for western blot analysis.

Finally, the rats were deeply anesthetized with 5% isoflurane, and the microdialysis probe was removed. A blood sample was collected from the heart, and a CSF sample was collected from the cisterna magna for the metabolomic analysis of neurotransmitters. Furthermore, samples of muscles surrounding the acupoints were collected for Western blot analysis (Figure 2).

### 2.6. Liquid Chromatography-ElectrosprayIonization-Tandem Mass Spectrometry Analysis for the Measurement of Neurotransmitters

Before analysis, 30 *μ*L of the extracellular fluid, serum, and CSF samples was completely mixed with 120 *μ*L of methanol through vigorous vertexing. The sample methanol mixture was centrifuged at 14,000 rpm for 10 min, and 120 *μ*L of the supernatant was collected for drying in a vacuum concentrator. The dried samples were dissolved in 50 *μ*L of ultrapure water, and 45 *μ*L of the supernatant was collected after centrifugation at 14,000 rpm for 10 min to measure the levels of neurotransmitters through liquid chromatography-electrosprayionization-tandem mass spectrometry (LC/ESI-MS/MS) by using the multiple reaction monitoring/electrospray ionization (ES)+ mode with the Xevo TQ XS system (Waters, Milford, USA, version 4.2).

Neurotransmitters were separated through reverse phase ultra-performance liquid chromatography (UPLC) on the Acquity UPLC BEH C18 1.7 *μ*m column (2.1 mm × 50 mm; Waters, Milford, USA) at 30°C. The elution was started using 99% mobile phase *A* (0.1% formic acid in ultrapure water) and 1% mobile phase *B* (0.1% formic acid in methanol), held at 1% *B* for 0.5 min, raised to 90% *B* in 2.5 min, held at 90% *B* for 0.5 min, and then lowered to 1% *B* in 0.5 min. The column was equilibrated by pumping 1% B for 2 min. The flow rate was set at 0.2 mL/min with an injection volume of 7.5 *μ*L. Mass spectra and chromatograms were acquired in the ES+ mode and processed using Mass Lynx software (Waters, Milford, USA). Neurotransmitters in each sample were determined using liquid chromatography retention plus tandem mass spectrometry and quantified on the basis of ion mass transitions.

### 2.7. Western Blot Analysis

The samples (acupoint muscles including the tibialis anterior muscle in ST36 and ST37 and flexor digitorum profundus muscle in PC6 and PC7 were homogenized at a ratio of 1 : 4 (w/v) in lysis buffer (50 mM Tris, pH 7.4; 250 mM NaCl, 5 mM EDTA; 50 mM NaF; 1 mM Na3VO4; 1% Nonidet P-40, 0.02% NaN_3_) containing a protease inhibitor cocktail. The homogenate was centrifuged at 12,000 rpm for 10 min at 4°C. The supernatant was collected to measure the protein concentration. Subsequently, 30 *μ*g of the sample was separated through 10% polyacrylamide gel electrophoresis and then blotted on a polyvinylidene fluoride membrane. The membrane was blocked with 5% nonfat milk in TBST buffer for 1 h and then washed with TBST for 5 min three times. The membrane was then incubated with primary antibodies diluted in phosphate buffered saline under shaking at 4°C overnight. Subsequently, the membrane was incubated with a secondary antibody (1 h at room temperature) under shaking and washed with TBST 5 min three times before and after the addition of the secondary antibody. Proteins were detected using an electrochemiluminescence kit according to the manufacturer's instructions.

The following primary antibodies were used: anti-P2X2 receptor (1 : 1000, Bioss, USA, lot: AB122401), anti-P2X3 receptor (1 : 1000, Millipore, USA, lot: 3690729), anti-transient receptor potential cation channel subfamily V member (TRPV) 4 receptor (1 : 1000, Alomone labs, Israel, lot: ACC034AN1202), anti-TRPV1 receptor (1 : 1000, Abcam, UK, lot: GR3392032 1), and anti-Piezo1/Piezo2 receptor (1 : 1000, Alomone labs, Israel, lot: APC087AN0550/APC090AN0150). The peroxidase conjugated antibody (1 : 500) was used as the secondary antibody. In addition, the following primary antibodies were used in the analysis of the acupoint muscle samples: tumor necrosis factor *α* (TNF *α*; 1 : 1000, Novusbio, USA, lot: 31050) and interleukin 10 receptors (1 : 1000, Invitrogen USA, lot: RA220898). All Western blot data were analyzed using AlphaEaseFC TM software, version 3.1.

### 2.8. Data Preparation and Statistical Analysis

Data are presented as the mean ± standard error. All statistical analyses were performed using SPSS. Significant differences among the five groups were analyzed using one-way analysis of variance, followed by the least significant difference post-hoc test. A *p* value of <0.05 indicated statistical significance.

## 3. Results

### 3.1. Effects of EA on Purinergic Receptor, Cytokines, and Mechanoreceptors: Western Blot Analysis

**Figure 3:**
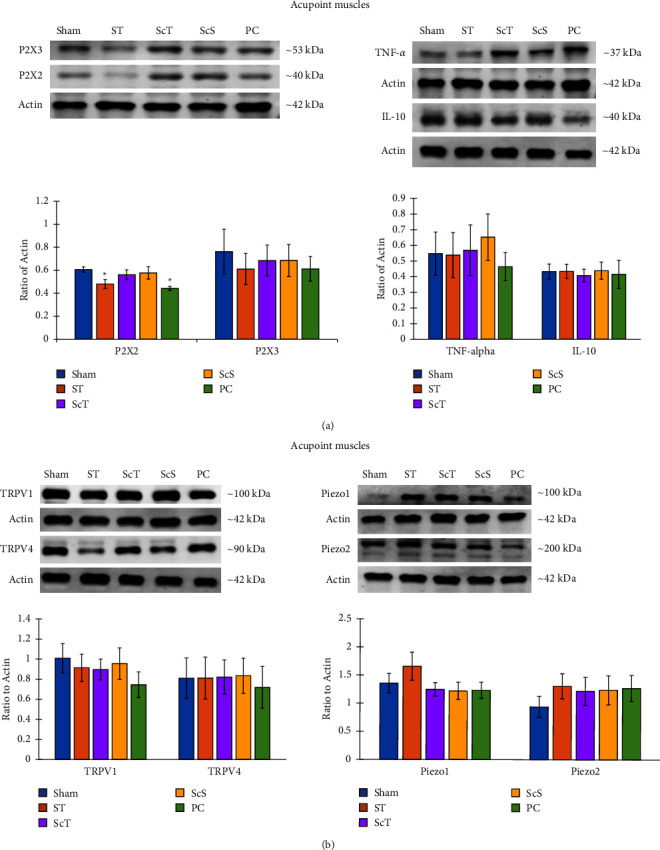
Effect of electroacupuncture on purinergic and mechanoreceptors in the acupoint muscles. (a) The P2X2 receptor expression was higher in the sham group than in the ST and PC groups, whereas the expression of the P2X3 receptor, tumor necrosis factor *α* (TNF *α*), and interleukin 10 (IL-10) did not significantly differ among the sham, ST, ScT, ScS, and PC groups; (b) the expression of receptor potential cation channel subfamily *V* member 4 (TRPV4), TRPV1, Piezo1, and Piezo2 receptors did not significantly differ among the sham, ST, ScT, ScS, and PC groups. Sham: sham group; ST: ST group; ScT: ScT group; ScS: ScS group; PC: PC group; ^*∗*^*p* < 0.05 compared with sham; *n* = 6.

The expression of the P2X2 receptor in the acupoint muscle tissues was stronger in the sham group than in the ST and PC groups (both *p* < 0.05; Figure 3(a)). However, the expression of the P2X2 receptor did not significantly differ among the sham, ScT, and ScS groups; between the ST and PC groups; and among the ST, ScT, ScS, and PC groups (all *p* > 0.05; Figure 3(a)). The expression of TNF *α* and IL 10 receptors in the acupoint muscle tissues did not significantly differ among the sham, ST, ScT, ScS, and PC groups (all *p* > 0.05; Figure 3(a)).

The expression of P2X3, TRPV1, TRPV4, Piezo1, and Piezo2 receptors in the acupoint muscle tissues did not significantly differ among the sham, ST, ScT, ScS, and PC groups (all *p* > 0.05; Figure 3(b)).

### 3.2. Effects of EA on Neurotransmitters in the Extracellular Fluid: Metabolomic Analysis

**Figure 4:**
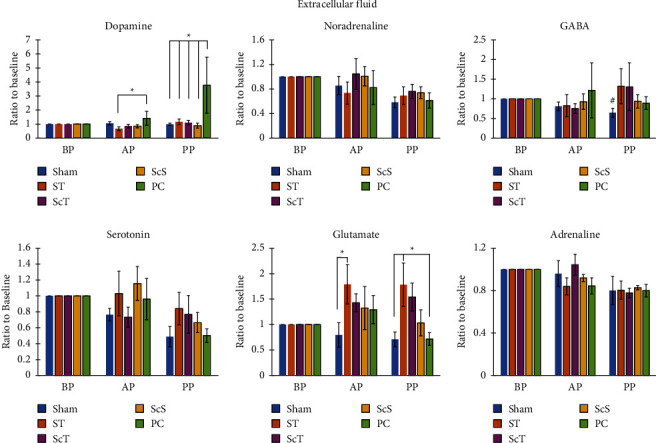
Effect of electroacupuncture on neurotransmitters in the extracellular fluid surrounding the acupoint. Dopamine levels in the extracellular fluid surrounding the acupoints were higher in the PC group than in the sham, ST, ScT, and ScS groups during the postacupuncture period and higher than the ST group during the acupuncture period. Glutamate levels in the extracellular fluid surrounding the acupoints were higher in the ST group than in the sham group during the acupuncture period and higher than in the sham and PC groups during the postacupuncture period. Sham: sham group; ST: ST group; ScT: ScT group; ScS: ScS group; BP: preacupuncture period; AP: acupuncture period; PP: postacupuncture period. ^*∗*^*p* < 0.05; *n* = 6.

Dopamine levels were similar in the extracellular fluid surrounding the acupoints among the sham, ST, ScT, ScS, and PC groups during the preacupuncture period (baseline; all *p* > 0.05; Figure 4). Dopamine levels in the extracellular fluid surrounding the acupoints were higher in the PC group than in the ST group during the acupuncture period (*p* < 0.05; Figure 4). However, dopamine levels were similar among the sham, ScT, ScS, and PC groups and among the sham, ST, ScT, and ScS groups (all *p* > 0.05; Figure 4). Dopamine levels in the extracellular fluid surrounding the acupoints during the postacupuncture period were higher in the PC group than in the sham, ScT, and ScS groups (all *p* < 0.05; Figure 4). However, dopamine levels were similar between the ST and PC groups and among the sham, ST, ScT, and ScS groups (all *p* > 0.05; Figure 4).

Noradrenaline levels in the extracellular fluid surrounding the acupoints were similar during the preacupuncture, acupuncture, and postacupuncture periods among the sham, ST, ScT, ScS, and PC groups (all *p* > 0.05; Figure 4).

*γ*-Aminobutyric acid (GABA) levels in the extracellular fluid surrounding the acupoints were similar during the preacupuncture, acupuncture, and postacupuncture periods among the sham, ST, ScT, ScS, and PC groups (all *p* > 0.05; Figure 4).

Serotonin levels in the extracellular fluid surrounding the acupoints were similar during the preacupuncture, acupuncture, and postacupuncture periods among the sham, ST, ScT, ScS, and PC groups (all *p* > 0.05; Figure 4).

Glutamate levels in the extracellular fluid surrounding the acupoints were similar among the sham, ST, ScT, ScS, and PC groups during the preacupuncture period (all *p* > 0.05; Figure 4). Glutamate levels were higher in the ST group than in the sham group during the acupuncture period (*p* < 0.05; Figure 4) but similar among the sham, ScT, ScS, and PC groups and among the ST, ScT, ScS, and PC groups (all *p* > 0.05; Figure 4). Furthermore, glutamate levels were higher in the ST group than in the sham and PC groups during the postacupuncture period (both *p* < 0.05; Figure 4) but similar among the sham, ScT, ScS, and PC groups and among the ST, ScT, and ScS groups (all *p* > 0.05; Figure 4).

Adrenaline levels in the extracellular fluid surrounding the acupoints were similar during the preacupuncture, acupuncture, and postacupuncture periods among the sham, ST, ScT, ScS, and PC groups (all *p* > 0.05; Figure 4).

### 3.3. Effects of EA on Neurotransmitters in Peripheral Blood: Metabolomic Analysis

**Figure 5:**
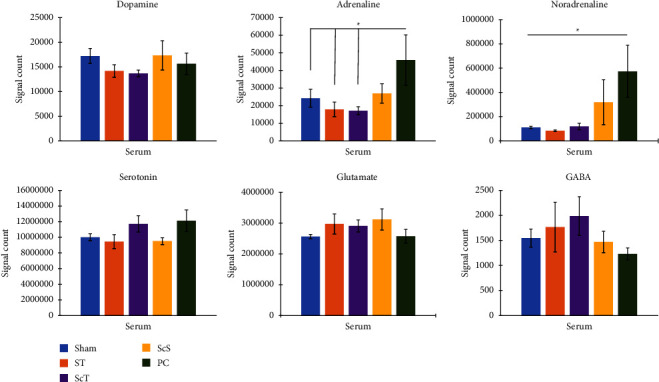
Effect of electroacupuncture on neurotransmitters in the peripheral blood. Serum adrenaline and noradrenaline levels were higher in the PC group than in the sham, ST, and ScT groups. Sham: sham group; ST: ST group; ScT: ScT group; ScS: ScS group; ^*∗*^*p* < 0.05; *n* = 6.

Serum dopamine levels did not significantly differ among the sham, ST, ScT, ScS, and PC groups (all *p* > 0.05; Figure 5).

Serum adrenaline levels were higher in the PC group than in the sham, ST, and ScT groups (all *p* < 0.05; Figure 5). Furthermore, serum adrenaline levels did not significantly differ among the sham, ST, ScT, and ScS groups and between the ScS and PC groups (all *p* > 0.05; Figure 5).

Serum noradrenaline levels were higher in the PC group than in the sham, ST, and ScT groups (all *p* < 0.05; Figure 5). However, serum noradrenaline levels did not significantly differ among the sham, ST, ScT, and ScS groups and between the ScS and PC groups (all *p* > 0.05; Figure 5).

Serum serotonin, glutamate, and GABA levels did not significantly differ among the sham, ST, ScT, ScS, and PC groups (all *p* > 0.05; Figure 5).

### 3.4. Effects of EA on Neurotransmitters in the CSF: Metabolomic Analysis

**Figure 6:**
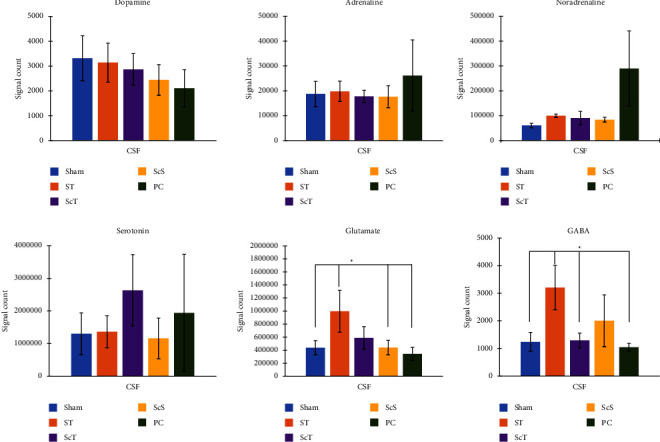
Effect of electroacupuncture on neurotransmitters in the cerebrospinal fluid (CSF). Glutamate levels in the CSF were higher in the ST group than in the sham, ScS, and PC groups. GABA levels in the CSF were higher in the ST group than in the sham, ScT, and PC groups. Sham: sham group; ST: ST group; ScT: ScT group; ScS: ScS group; ^*∗*^*p* < 0.05; *n* = 6.

The dopamine, adrenaline, noradrenaline, and serotonin levels in the CSF did not significantly differ among the sham, ST, ScT, ScS, and PC groups (all *p* > 0.05; Figure 6).

Glutamate levels in the CSF were higher in the ST group than in the sham, ScS, and PC groups (all *p* < 0.05; Figure 6). However, glutamate levels in the CSF did not significantly differ among the sham, ScT, ScS, and PC groups and between the ST and ScT groups (all *p* > 0.05; Figure 6).

GABA levels in the CSF were higher in the ST group than in the sham, ScT, and PC groups (all *p* < 0.05; Figure 6), whereas GABA levels in the CSF did not significantly differ among the sham, ScT, ScS, and PC groups and between the ST and ScS groups (all *p* > 0.05; Figure 6).

The aforementioned results are summarized in Tables 1 and 2.

## 4. Discussion

The results of the present study revealed that the expression of P2X2 receptors was stronger in the sham group than in the ST and PC groups and did not significantly differ between the sham and ScT groups. Dopamine levels in the extracellular fluid surrounding the acupoints were higher in the PC group than in the sham and ST groups during the postacupuncture period. Glutamate levels in the extracellular fluid surrounding the acupoints were higher in the ST group than in the sham group during the acupuncture period and higher in the ST group than in the sham and PC groups during the postacupuncture period. Furthermore, serum adrenaline and noradrenaline levels were higher in the PC group than in the sham, ST, and ScT groups. Glutamate levels in the CSF were higher in the ST group than in the sham, ScS, and PC groups, and GABA levels in the CSF were higher in the ST group than in the sham, ScT, and PC groups. The insertion of an acupuncture needle into an acupoint through the skin causes the release of ATP from keratinocytes and fibroblasts; the released ATP then binds to its P2X2 and P2X3 receptors expressed on free nerve endings. Subsequently, the acupuncture signal is transmitted from the peripheral nerve and spinal cord to the brain [7, 14]. The P2X2 and P2X3 receptors are widely expressed on nociceptive sensory neurons and play a vital role in pain transduction. These subunits can form homotrimeric P2X2, P2X3, or heterotrimeric P2X2/3 receptors. Each of these receptor subtypes might mediate different pathological pain conditions. The activation of P2X3 leads to rapid desensitization, the stimulation of P2X2 leads to a sustained current, and the activation of P2X2/3 heterotrimers causes intermediate desensitization [15]. A study [16] demonstrated that the intra-arterial infusion of *α*, *β* methylene ATP (a selective P2X receptor agonist) caused localized vasoconstriction in the experimental arm. Activation of P2X receptors in the arterial vasculature of the skeletal muscle caused vasoconstriction in both resting and active skeletal muscles [16]. These results suggest that EA inhibits the fast synaptic transmission of P2X2 receptors, reducing the vasoconstriction of the skeletal muscle and thus alleviating pain. The results of the present study indicated that the bilateral neurectomy of the sciatic nerve reversed the expression of P2X2 receptors reduced by EA at ST36 and ST37. This result is consistent with the finding that acupuncture signals from P2X2 receptors are transmitted from the sensory nerve to the spinal cord and higher centers [7, 14]. Taken together, the study initially revoked the capable application of 2 Hz EA at those acupoints for pain relieving treatment.

Our results revealed that the expression of TNF *α*, IL-10, TRPV1, TRPV4, Piezo1, and Piezo2 did not significantly differ among the sham, ST, ScT, ScS, and PC groups. TNF *α* is a proinflammatory cytokine produced by macrophages in response to inflammation, infection, or tissue damage [17]. IL-10 plays an anti-inflammatory role and inhibits the production of proinflammatory cytokines, such as IL 1*β* and TNF *α*. IL-10 is produced by both immune cells, including T cells, macrophages, and dendritic cells, and nonimmune cells, such as keratinocytes and epithelial cells [18, 19]. The TRPV1 receptor can be activated by capsaicin, nociceptive heat (≥43°C), and camphor and induces pain in the presence of thermal stimuli. Furthermore, the TRPV1 receptor is expressed on the terminals of the small diameter afferent sensory neurons, dorsal root ganglion, spinal cord, and brain [20, 21]. TRPV4 plays the role of a transducer in pain caused by osmolarity changes [22]. In addition, TRPV4 can act as a transducer of warm stimuli in the anterior hypothalamus [23]. The Piezo ion channel is necessary in mechanical transduction processes, such as light touch recognition, proprioception, and vascular blood flow. Piezo1 can regulate red cell volume and shear stress, and Piezo2 is expressed on Merkle cells in response to tough stimulation. Moreover, Piezo2 is required for Merkel cell mechanosensitivity [24, 25]. Taken together, the relationship between the application of EA and the expression of TNF *α*, IL-10, TRPV1, TRPV4, Piezo1, and Piezo2 should be investigated in future studies.

Glutamate is an excitatory neurotransmitter present in the brain and spinal cord and plays a critical role in neuronal cell differentiation, growth, migration, and survival. Glutamate is involved in many neurological functions, including cognition, memory, neurological network formation, and behavior [26, 27]. GABA is a major inhibitory neurotransmitter in the central nervous system, and it regulates the sleep cycle and prevents seizures. GABA is present in different brain regions, including the amygdala, hippocampus, hypothalamus, and prefrontal cortex, and modulates excitation and neural synchronization [28]. The activation of the GABA_A_ receptor reduced neuronal death and provided neuroprotection in focal ischemia mice and rats [29, 30]. In Traditional Chinese Medicine (TCM), acupuncture at both ST36 and ST37 belonging to the stomach meridian is applied to treat digestive system disorders, such as abdominal pain and distension [31]. Moreover, acupuncture at ST36 increased motor cortical excitation and reduced motor cortical inhibition [32]. Our previous study reported that the application of EA at bilateral ST36 and ST37 increased the P25 amplitude of somatosensory evoked potentials and inhibited skin sympathetic responses, thus causing cerebral modulation [33]. Acupuncture at ST36 transports substances possibly through a primitive vascular-like system that links between the skin and brain to promote neurogenesis [34]. Taken together, the results suggest that the application of EA at bilateral ST36 and ST37 modulates the excitation of the cerebral cortex, enhances neurological function, and provides neuroprotection. Glutamate from the presynaptic neuron released into the synaptic cleft and combines with *N* methyl-D-aspartic acid (NMDA) and 2-amino-3-(3-hydroxy-5-methyl-isoxazol-4-yl) propanoic acid (AMPA) receptors of postsynaptic neurons can cause the generation of long-term potentiation in the central nervous system. Long-term potentiation plays a critical role in memory and cognitive function, whereas GABA inhibits the propagation of action potential [35]. In addition, metabotropic GABA B receptors can directly affect different types of postsynaptic glutamic receptors to modulate the excitability of circuits in brain. In contrast, NMDA receptor activity can also modulate GABAB receptors expression [36], therefore, both glutamate and GABA B play a role of each other's regulation.

Dopamine in the brain mainly originates from dopaminergic neurons in the substantia nigra and ventral tegmental area and regulates cognition, locomotor control, and emotion [37, 38]. Peripheral dopamine mainly results from amine precursor uptake and the decarboxylation of cells in the sympathetic nervous system and adrenal glands in response to environmental stress, such as that induced by a high fat diet [37]. Dopamine can reduce renal vascular resistance and increase renal blood and cardiac output in patients with heart failure [39]. Adrenaline can increase left ventricle contraction and cardiac output [40]. Noradrenaline can increase the heart rate and reduce atrioventricular conduction to increase the strength of cardiac contraction, and adrenaline can affect the cardiac muscle and peripheral vessels [41]. In addition, circulating adrenaline causes large increases in blood glucose and energy substrate levels in the skeletal muscles, heart, and brain during a sympathetic response [42]. Acupuncture at both PC6 and PC7 belonging to the pericardium meridian is applied to treat cardiovascular diseases, such as cardiac pain and palpitation, in TCM [31]. Furthermore, the application of EA at PC6 ameliorated myocardial ischemia (MI) in an ischemic heart rat model by mediating the activation of A1 and A2b adenosine receptors [43]. Acupuncture at PC6 increased left ventricular systolic and diastolic function in rats with isoproterenol-induced MI [44]. Taken together, the application of EA at PC6 and PC7 can enhance heart function. Both dopamine and noradrenaline have a vasopressor effect and are used as first-line agents to correct hypotension in patients with circulatory shock; dopamine is weaker than noradrenaline in vasopressor effect but can increase cardiac output more than noradrenaline. In addition, dopamine can increase global blood flow including renal and hepatosplanchnic blood flow [45].

In TCM, acupuncture is mainly used to treat various diseases in accordance with the principle of meridian theory. For instance, acupuncture at PC6 belonging to the pericardium meridian can treat cardiovascular disease. Similarly, acupuncture at ST36 belonging to the stomach meridian can treat gastric disorders [46]. The results of the present study demonstrated that application of 2 Hz EA at PC6 and PC7 increased dopamine levels and serum adrenaline and noradrenaline levels in the extracellular fluid surrounding the acupoints. These results support meridian theory by indicating that acupuncture at PC6 can treat cardiovascular disease. Moreover, the findings of the present study indicated that the application of 2 Hz EA at ST36 and ST37 increased glutamate levels in the extracellular fluid surrounding the acupoints and glutamate and GABA levels in the CSF. Acupuncture signal from acupoint and from splanchnic organs is integrated in the central nervous system [46, 47]. Moreover, the results suggest that circulating neurotransmitters are involved in transmitting acupuncture signals.

Because neurotransmitter has a clear physiological function of body organs including the heart and nervous system, the present study chooses neurotransmitters, and the present study also defines acupoint specificity that acupuncture at the acupoint can produce neurotransmitter changes related to the physiological function of the acupoint. The present study lacks direct detect painful behaviors as well as heart and brain function, so it can only be said to be an observation of a phenomenon, not direct evidence that EA could modulate pain, heart, and brain function through these receptors and neurotransmitters. Furthermore, study is needed in the future.

Several studies report that isoflurane can inhibit mitochondria complex I or presynaptic Ca^2+^ influx to inhibit synaptic vesicle exocytosis at nerve terminals. These effects of isoflurane can affect neurotransmitter release including glutamate and GABA [48, 49]. In addition, the rats were operated, the microdialysis probes were implanted, and acupuncture needles were inserted into muscle tissues. These procedures were all invasive, all of which may affect possibly the relevant neurotransmitters. Therefore, how to correct for these factors are an important issue. In this study, we deigned a sham group without EA, and acupuncture needles were only inserted into the subcutaneous layer between ST36 and ST37. The extracellular fluids were collected in the preacupuncture, acupuncture, and postacupuncture periods, and that was similar to the ST and PC groups as possible as to reduce the bias. However, how to seek an anesthetic that does not affect neurotransmitters, use smaller microdialysis probes, and acupuncture that minimizes tissue damage such as laser acupuncture in the future study.

## 5. Conclusion

Application of 2 Hz-EA at ST36 and ST37 and PC6 and PC7 reduced the expression of P2X2 receptors in the acupoint muscles, and this effect of EA at ST36 and ST37 could be reversed through the neurectomy of the bilateral sciatic nerves, indicating that the analgesic effect of EA at ST36 and ST37 and PC6 and PC7 is related to sensory afferent nerve transmission. Moreover, the application of 2 Hz-EA at PC6 and PC7 increased dopamine levels in the extracellular fluid surrounding the acupoints and serum adrenalin and noradrenalin levels, indicating that EA at PC6 and PC7 can enhance heart function. In addition, the application of 2 Hz-EA at ST36 and ST37 increased glutamate levels in the extracellular fluid surrounding the acupoints and glutamate and GABA levels in the CSF, indicating that EA at ST36 and ST37 modulates the cerebral cortex to promote and protect neurological function. These results are consistent with meridian theory in TCM. However, the present study lacks direct detect painful behaviors as well as heart and brain function, further study is needed in the future.

As this present study is the first to investigate the effects of EA on alterations of neurotransmitters that might be associated with its physiological functions, extensive research is needed to clarify whether EA can indeed result in analgesic effects and effects on other organs, such as heart and kidney functions. In the same way, we are conducting follow-up research with more critical outcomes that will prove the effects of EA.

## Figures and Tables

**Table 1:** Effect of electroacupuncture on the acupoint muscles, surrounding acupoint extracellular fluid, serum, and cerebrospinal fluid.

	Sham	ST	ScT	ScS	PC
Acupoint muscles					
P2X2 receptor		Decrease	—	—	Decrease
Extracellular fluid					
Dopamine		—	—	—	Increase
Glutamate		Increase	—	—	—
Serum					
Adrenaline		—	—	—	Increase
Noradrenaline		—	—	—	Increase
Cerebrospinal fluid					
Glutamate		Increase	—	—	—
GABA		Increase	—	—	—

The results were in comparison with sham; the levels were not significantly different compared with sham. Sham: sham group; ST: St group; ScT: ScT group; ScS: ScS group; PC: PC group; GABA: *γ* aminobutyric acid; decrease: *p* < 0.05; increase: *p* < 0.05.

**Table 2:** Physiological function of protein and neurotransmitters.

Protein and neurotransmitters	Physiological functions
P2X2 receptor	(1) A receptor of ATP
	(2) Pain transduction

Dopamine	(1) Regulates cognition, locomotor control, and emotion
	(2) Response to environmental stress
	(3) Increases renal blood and cardiac output

Adrenaline	(1) Increase left ventricle contraction and cardiac output
	(2) Increases in blood glucose and energy substrate levels in the skeletal muscles, heart, and brain during a sympathetic response

Noradrenaline	(1) Increase the heart rate and reduce atrioventricular conduction to increase the strength of cardiac contraction

Glutamate	(1) An excitatory neurotransmitter
	(2) Neuronal cell differentiation, growth, migration, and survival
	(3) Cognition, memory, neurological network formation, and behavior

GABA	(1) Inhibitory neurotransmitter
	(2) Regulates the sleep cycle and prevents seizures
	(3) Modulates excitation and neural synchronization

ATP: adenosine triphosphate; GABA: *γ* aminobutyric acid.

## Data Availability

The data supporting the findings of the study are available from the corresponding authors upon request.
